# A Comprehensive Analysis: Evaluating Security Characteristics of Xbee Devices against Zigbee Protocol

**DOI:** 10.3390/s23218736

**Published:** 2023-10-26

**Authors:** Vlad Gavra, Ovidiu A. Pop, Ionut Dobra

**Affiliations:** 1 Faculty of Electronics, Telecommunications and Information Technology, Technical University of Cluj-Napoca, 400114 Cluj-Napoca, Romania; vlad.gavra@yahoo.com (V.G.); ovidiu.pop@ael.utcluj.ro (O.A.P.); 2 Faculty of Automatic Control and Computer Science, Technical University of Cluj-Napoca, 400114 Cluj-Napoca, Romania

**Keywords:** Zigbee, Xbee, security, Zigbee keys, digital signature, Zigbee security models

## Abstract

In recent times, the security of sensor networks, especially in the field of IoT, has become a priority. This article focuses on the security features of the Zigbee protocol in Xbee devices developed by Digi International, specifically in the Xbee 3 (XB3-24) devices. Using the TI LaunchXL-CC26X2R1 kit, we intercepted and analyzed packets in real-time using the Wireshark application. The study encompasses various stages of network formation, packet transmission and analysis of security key usage, considering scenarios as follows: without security, distributed security mode and centralized security mode. Our findings highlight the differences in security features of Xbee devices compared to the Zigbee protocol, validating and invalidating methods of establishing security keys, vulnerabilities, strengths, and recommended security measures. We also discovered that security features of the Xbee 3 devices are built around a global link key preconfigured therefore constituting a vulnerability, making those devices suitable for man-in-the-middle and reply attacks. This work not only elucidates the complexities of Zigbee security in Xbee devices but also provides direction for future research for authentication methods using asymmetric encryption algorithms such as digital signature based on RSA and ECDSA.

## 1. Introduction

Technology is based on a wide range of sensors used for various applications: there are different sensors for industry, air quality, smart public transportation, smart grids, and so on. All these devices have one thing in common, which is at the same time their most important feature, and that is communication. In order to make sensors easier to use and to connect within automation processes, connection between devices should be wireless, and for this purpose, ZigBee technology is one of the most sustainable solutions. The purpose of wireless communication is to collect data or to execute a specific task in the network without putting too much effort in the installation step of the device, such as wiring. ZigBee protocol enhances the IEEE 802.15.4 standard [1] by adding ZigBee layers on top of the IEEE 802.15.4 layers consisting in network layer and application layer together with security services [2,3,4,5].

Some of the ZigBee protocol characteristics:

- Global operation frequency at 2.4 GHz band specific of IEEE 802.15.4 standard;
- Low-power consumption;
- Discovery mechanism with full application confirmation;
- Pairing mechanism with full application confirmation;
- Various transmission options including broadcast;
- Mechanism for security key generation;
- AES-128 standard security scheme.

## 2. Previous Work

There are several papers that study the security of the Zigbee protocol under conditions such as attacks that can be launched on Zigbee-based communication devices. The authors of the paper [6] launched attacks such as key sniffing, association flooding, and replay attacks using the Atmel Raven RZUSB Stick for message interception and the Wireshark application for analysis of intercepted messages. The first attack launched was the network key sniffing attack launched when the network was formed. The network key was encrypted in this case with the default TC link key. Since the link key is the default, one the authors found it very easy to discover the network key used. Based on the first attack launched, the two subsequent attacks were also launched, which were unsuccessful due to the other Zigbee security features.

In paper [7], the authors started from the same premise of discovering the encryption key, the method of acquisition being different in this case. To discover the encryption key, the memory of a device in the network was read so that the key was discovered unencrypted. Attacks such as DOS and Replay were launched: both attacks were successfully launched. We can see that in both papers presented security is performed at a low level, relying on default keys or even not being present at all. In order to make use of the security offered by the Zigbee protocol, this paper proposes a detailed analysis of the Zigbee security features present in Digi International’s Xbee 3 devices.

## 3. Overview of ZigBee Protocol

ZigBee was developed by ZigBee Alliance, formed by many big companies such as Freescale, Chipcon, Mitsubishi, NXP Semiconductors, and Texas Instruments. ZigBee is a LP-WPAN (Low-Power-Wireless Personal Area Network) with short range and low power consumption, as mentioned before. The range for ZigBee devices is up to fifty meters and it is characterized by low data rate, having a maximum value of 250 kbps. The protocol is suitable for sensors and IoT applications because of the low data rate and low power consumption, being able to power sensors from batteries for months or even up to years [2,3,4]. IEEE 802.15.4 standard provides 64-bit and 16-bit short addresses supporting, in theory, more than 65,000 nodes per network; based on this capability and the range of ZigBee devices, applications can be scaled to multiple sensors, covering wide areas of space.

Some of the ZigBee protocol characteristics:

- Global operation frequency at 2.4 GHz band specific of IEEE 802.15.4 standard;
- Low-power consumption which extends battery life;
- Low cost;
- Open standard protocol;
- Secured protocol;
- Discovery mechanism with full application confirmation;
- Pairing mechanism with full application confirmation;
- Various transmission options including broadcast;
- Mechanism for security key generation;
- AES-128 standard security scheme;
- Over the air firmware upgrade.

## 4. ZigBee Protocol Architecture

**Figure 1:**
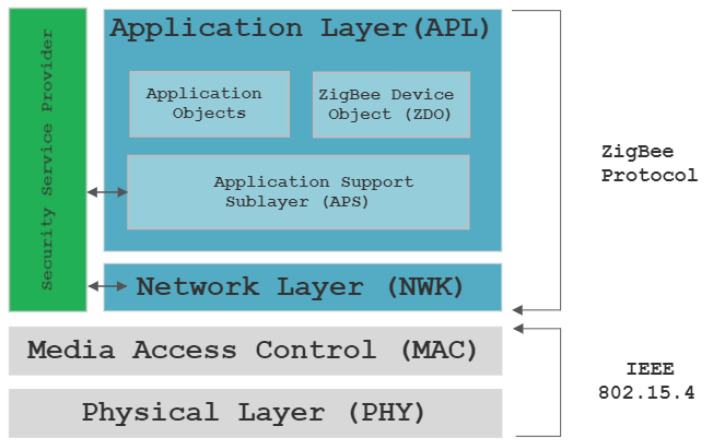
ZigBee Protocol Layers.

The ZigBee protocol architecture is composed of several layers built over IEEE 802.15.4 standard, which is characterized by physical layer (PHY) and media access control layer (MAC), complementing the standard mentioned before with network layer (NWK), application layer (APL), and security service provider. Application layer comprises the following sublayers: Application objects, ZigBee device object (ZDO), and Application support sublayer (presented in Figure 1). Together, these layers assure the functionality of the ZigBee protocol, as well as data integrity and security and some predefined functions, in order to offer possibilities for applications to use different manufacturers for devices and still being able to execute tasks in the same network without any customization effort [8,9,10,11].

Physical layer (PHY)—This layer of the IEEE 802.15.4 standard is closely related to the hardware and defines physical operations for ZigBee devices as modulation, demodulation, initialization of the hardware, channel selection, energy detection measurement, physical transmission of packets, and different techniques and mechanisms to avoid radio noise transmission interference.

Media Access Control layer (MAC)—This layer is also defined by IEEE 802.15.4 standard and constitutes a bridge between physical and network layers and provides two services—MAC data services and MAC management service. In addition, this layer defines four frame structures: Beacon frame, Data frame, Acknowledge frame, and MAC command frame. The main purpose of this layer is to identify network topologies and to avoid collisions during frame transmission using CSMA-CA protocol.

Network Layer (NWK)—This layer is part of the ZigBee protocol and comes above MAC layer defined by IEEE 802.15.4 standard, and it is responsible for routing the packages inside the network by crossing multiple equipment before reaching destination. Based on this information, the Network layer manages neighbors discovering, routes discovering, routes maintenance, and joining or leaving network mechanism.

Application layer (APL)—This layer is the highest layer form ZigBee protocol and it hosts the applications objects. APL layer is sub divided in three sub-layers: Application objects, ZigBee Device Object, and Application Support Sublayer.

- Application object;
- ZigBee device object;
- Application support sublayer.

Security service provider—This layer is part of the ZigBee protocol and provides security services for both NWK and APS layers used for device management, frame security, methods for key establishment and key transportation.

## 5. Zigbee Frame Format and Encapsulation

**Figure 2:**
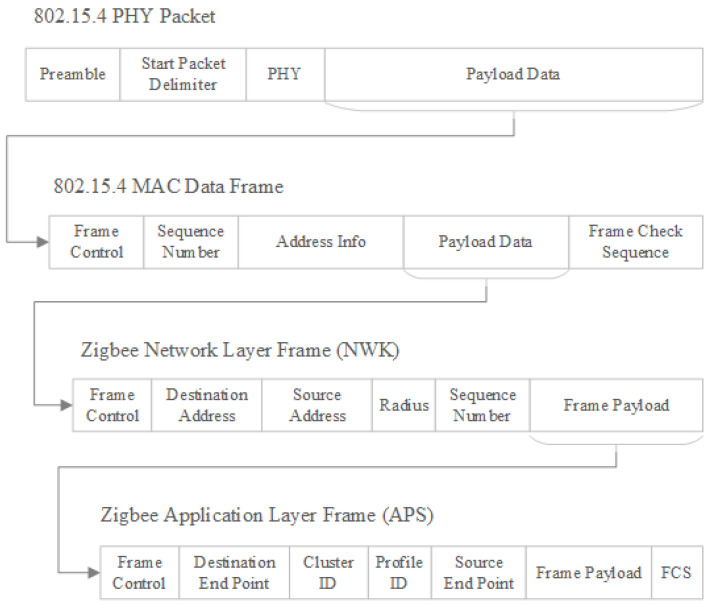
ZigBee frame encapsulation according to protocol architecture.

Zigbee is forming its frames based on the architecture presented in Figure 1. Starting from the top layer of Zigbee protocol, Application layer, frames are created and encapsulated as payload of the next layer frame up to the last layer of the architecture. Zigbee frame formation is presented in Figure 2 [9,11].

The purpose of the APS layer is to provide an interface between application and network. The Zigbee frame at the application layer includes the APS header, APS payload and APS footer, where APS header contains control information, destination and source endpoints, cluster ID, profile ID, while APS footer contains a frame check sequence for error detection.

At the network layer, responsibility is oriented to network setup, end device joining, routing and device discovery. Components of the frame at this level are the same as they are at the previous layer, but in the payload, APS frame is encapsulated. The network header contains information such as frame control field (which contains a field to signal if security is activated), destination, and source addresses and sequence number.

The MAC layer manages access to the physical medium; frame structure includes MAC header, MAC payload, and MAC footer, where MAC Payload contains frame from previous layer.

PHY Layer is the lowest level used to transmit and receive raw bit streams over the physical medium and, in addition to MAC frame data, PHY frame contains synchronization header and sequence delimiter which announce the start of the actual data.

The MAC layer manages access to the physical medium; frame structure includes MAC header, MAC payload, and MAC footer, where MAC Payload contains frame from previous layer.

PHY Layer is the lowest level used to transmit and receive raw bit streams over the physical medium and, in addition to MAC frame data, PHY frame contains synchronization header and sequence delimiter which announce the start of the actual data.

## 6. ZigBee Security Architecture

As with any wireless communication protocol, ensuring secure data transmission is a crucial consideration, which the Zigbee protocol is fulfilling by applying several security features as encryption, using AES-128 algorithm, message integrity code applied to each packet and reply protection by using a sequence number and time freshness checks to prevent replay attacks. Those security measures, if they are implemented correctly, can make Zigbee networks highly secure. However, as with any technology, security is dependent on how it is implemented and maintained [1,12,13,14,15].

### 6.1. IEEE 802.15.4 Security

All the security features from this standard are handled in the MAC layer below application control. Security services provided on this level are: data confidentiality, message integrity, and protection against reply attacks. IEEE 802.15.4 specifications introduce procedures and mechanisms for protecting MAC frames through symmetric key cryptographic algorithms based on AES-CCM. This layer defines eight security levels to protect the frames generated by MAC which are presented in Table 1.

A MAC frame is composed of many different parts, but the most important part for this paper is the data package with the flag field which indicates if security is enabled, what addressing mode is in use, and whether the sender requests an acknowledgement. If the security flag is set to 0, then no security is active, but if the flag is set to 1, then one of the seven levels of security is active [1].

Categories that support data authenticity are available in three variants, depending on the message integrity code (MIC) length which can be 4, 8, of 16 bytes. The longer the message integrity code, the better protection against authenticity attacks. Together with data authenticity, IEEE standard also offers data confidentiality and replay protection. All the parameters related to security features can be configured in Auxiliary security header (ASH) from MAC Frame structure. ASH is formed of 14 bytes and composed of three fields as follows [16]:

- Security control header (1 byte);
- Frame counter (4 bytes)—responsible for protection against replay attacks;
- The key identifier (0–9 bytes)—containing key (0/4/8 bytes) and key index (1 byte) responsible for determination of the key for the encryption; this field is optional.

Auxiliary security header is transmitted in clear text and with authentication provided, if this is activated. Based on security possibilities data fields are formatted as follows: when AES-CTR is active then only payload of MAC frame is encrypted, when data authentication (MIC XX) is active then MAC frame header and MAC frame payload will be part of the data that the message integrity code will be generated on. When data confidentiality is also active, MAC frame payload and MIC are encrypted but encryption is carried out after MIC is calculated, as mentioned above. Even though security features are also provided at this layer, Zigbee protocol is not using any of those, security being assured at upper layers only [17,18,19].

### 6.2. ZigBee Stack Layers Security Architecture

Above PHY and MAC layer, ZigBee stack introduces two extra security levels through network layer and application layer. Security measures implemented by Zigbee standard are complex and intended to ensure key setting, secure networks, key transportation, and frame security at all levels layers. The Zigbee standard brings two network architectures, such as distributed and centralized security models, to satisfy a wide range of applications.

- The distributed security model—This model supports only routers and end devices, routers being part of this type of network and are responsible for the enrollment of new routers and end devices. If a router is sensing a powered up existing network, it may join the secured network, otherwise, a router can form a distributed secured network and issue network keys to newly joined routers and end devices. This model is simple but does not provide the best security.
- The centralized security model—This model is complex and more secured than the distributed model and brings into discussion another device—the trust center (network coordinator). The trust center is responsible for authentication and validation of each device which attempts to join the network.

### 6.3. Zigbee Security Keys

All the security policies rely on the AES 128 bits encryption algorithm, so the hardware architecture previously deployed for the link level is still valid. There are three kinds of keys, as follow [14]:

- Trust Center Link key—A secret key that is used by the network’s Trust Center to transmit Network Keys and Link keys in a secured manner. The TC Link key is only known to the Trust Center; those keys are pre-installed in each device by the manufacturer and are used to ensure the security of the ZigBee network by securing the link keys exchange between two nodes in the key establishment procedure.
- Link key—A unique key that is generated between two devices in a ZigBee network when they establish a secure connection. The Link Key is used to encrypt and decrypt messages between the two devices, and it is only known to those devices.
- Network key—A preshared 128-bit secret key that is used to secure communication between all devices in a ZigBee network. Devices in the network share the same Network Key and it is used to encrypt and decrypt messages sent between devices. This key is regenerated by Trust Center at different intervals.

### 6.4. ZigBee Stack—Network Layer Security

At Network layer security is pretty much the same as it is at MAC layer level, providing data confidentiality and authentication using AES-CCM. AES-CCM mode is an authenticated encryption algorithm designed to provide authentication and confidentiality during data transfer, combining counter mode encryption and CBC-MAC authentication mode. The key applied to AES-CCM algorithm is the network key, a 16-byte key, shared to all devices connected in the network. Every device that is accepted in the network should have a copy of the network key. In the centralized network, the trust center generates and stores multiple network keys, but only one is active at a time. The network key has a sequence number which is incremented every time when the network key is updated in the Zigbee network. The sequence number ranges from 0 to 255 and when it reaches the maximum number, 255, it wraps back to 0. An updated network key is usually sent to Zigbee devices encrypted with the link key when the link key is used. When a distributed TC security model is configured, and link keys are not configured or used, a network key is sent to Zigbee devices in clear text. It is important for devices in possession of the network key to store it securely [17,18,19].

**Figure 3:**
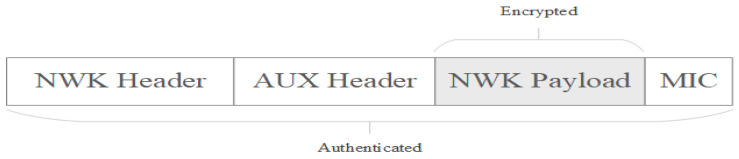
Secured frame format at Network layer.

The most important thing that has to be noted is that security at the Network layer in Zigbee protocol is carried out on a hop-by-hop basis, which means that each router that relays an encrypted packet first verifies that a valid encryption was carried out before going further with any other processing. The router checks the package by decrypting it and checking the message integrity code, then it re-encrypts the packet with its own network parameters, such as source address and frame counter, before sending the message to the next hop. This security feature is used to protect the network against attempts to inject bad traffic into the network and thus consuming network resources, transmitting packets which cannot be decrypted or used in the network. A packet secured at network layer is presented in Figure 3, having the following components: NWK Header, AUX Header, NWK Payload, and MIC [20,21].

The auxiliary header (AUX Header) is the element within the Network layer frame that provides information for a receiving node to accurately authenticate and decrypt the packet. These data include the type of key used, the sequence number, the IEEE address of the device that secured the data, and the frame counter used against reply attacks. Auxiliary header also includes a frame counter for every device from the network as a measure against reply attacks. Devices maintain a list of their neighbor’s and children’s frame counter and when the devices receive a frame from a neighbor it checks that the frame counter is higher than the last value stored in the table. If the values of the frame counter are increased, then the value is updated in the table, otherwise the packet is silently discarded. The frame counter is 32 bits long and may not wrap to zero. Every time the network key is updated, the frame counter may be reset to zero if the local device’s value is above 0x80000000.

Zigbee uses a symmetric key with a length of 128 bits for encrypting data transmitted at the network layer. The network header and the auxiliary header are transmitted in plain text, but they are considered when generating the message authentication code, while the payload data are both authenticated and encrypted. The AES-128 algorithm is used to create a 4-byte-long hash for the entire message created at the network layer, both for the header and for the payload data. The hash mentioned before is known as Message Integrity Code, MIC, and is used to authenticate the message by ensuring it has not been modified. A receiving device decrypts payload and calculates hash value of the received packet MIC and compares it with the original sent in the packet; if the values are not the same, the device will invalidate the packet entirely [21,22].

### 6.5. ZigBe Stack—Application Support Layer Security

APS security aims to offer end-to-end encryption to secure transmission of messages within a Zigbee network, ensuring that only the sender and recipient can decrypt the data. This is in contrast with network security layer, which offers security on a hop-by-hop basis. APS security uses a shared peer-to-peer key, named link key, that only the source and destination know about, establishing a secure connection between every Zigbee device in the application support layer [21].

There are two types of link keys used at Application layer—trust center link keys and application link keys.

**Figure 4:**
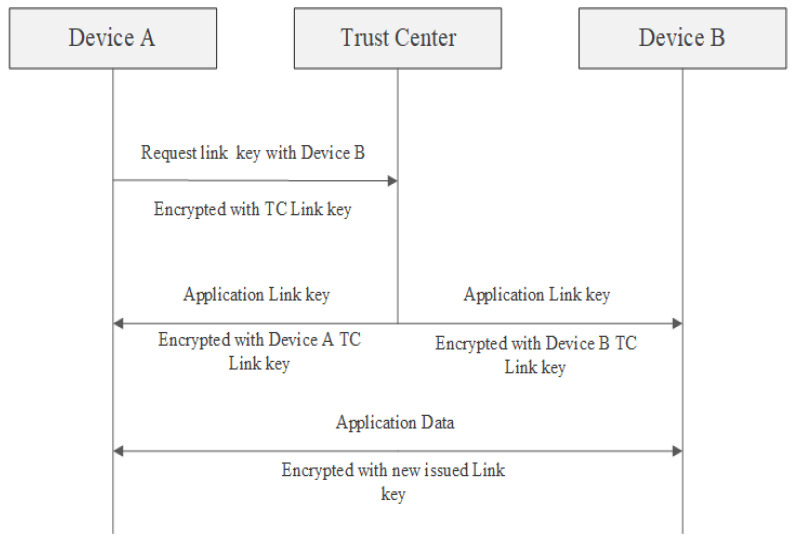
Application link key establishment method [23].

1. Trust center link key is configured between any device from the network and the Trust Center and it is used to secure APS command messages to and from the trust center and also by the application to encrypt data exchange.
2. Application link keys are shared between devices from the network where neither of the devices is a trust center and they may be used to add additional security to messages to or from an application running node. Because one key is used for only a pair of devices, a device can have multiple keys issued, depending on the number of devices that its communicating with. Application link keys can be either preconfigured or issued by the trust center after a request is sent to the trust center with this purpose. In the latter case, the trust center acts like a third party and sends the key, for a pair of devices, encrypted with trust center link key. Application link key establishment method is presented in Figure 4.

The trust center link key is used in the following cases: encrypting the initial transfer of the network key to joining node, encrypting an updated copy of the network key to a rejoining node that does not have the current key, routers sending or receiving APS messages to or from the trust center, application unicast messages to or from trust center.

Zigbee protocol offers another alternative to issue trust center link keys based on install codes, feature available only with Zigbee 3.0. Install codes key is just a preconfigured trust center link key used to enter the Zigbee network and obtain the current network key. Since both the device attempting to join and the trust center need to use this unique key during network entry, a set of sharable data known as the “installation code” used to derive the key should be known at both sides. The install code is used as the input to a Matyas–Meyer–Oseas (MMO) hash function, generating a hash value of a length of 128 bits. Hash value resulted is used as a preconfigured trust center link key for that device, and the trust center can then install a key table entry whit that key and the EUI64 of the joining device, which then allows the authentication to take place successfully during joining, and the joining device can successfully receive and decrypt the network key delivery.

**Figure 5:**
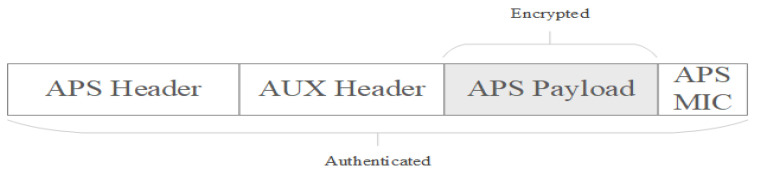
Secured frame format at Application Support layer.

Both APS layer and Network layer encryption can be used simultaneously to encrypt the contents of a message, in this case APS layer security is applied first, the frame created at APS is encapsulated as payload of the Network layer frame as in Figure 2, and encrypted after at Network layer. APS layer frame format is presented in Figure 5 [18,21].

When broadcast communication is secured using the network key, encrypted frames are passed to the network layer by the application support layer. When unicast communication requires security, the frames are encrypted with link keys in the application support layer. Each pair of Zigbee devices in the network shares a 128-bit link key exclusively. In addition, the application support layer is responsible for delivering key establishment, key transport, and device management services to applications and the Zigbee device object.

### 6.6. ZigBee Stack—Other Security Features

In addition to the security measures found in the network layer and the application support sublayer, Zigbee offers additional security functionalities. Among these is the adoption of application profiles. These profiles act as standardized protocols detailing message structures and processing procedures, allowing developers to craft applications that work seamlessly across devices from different manufacturers. Such profiles ensure that devices from diverse vendors can interact both effectively and securely.

Another security attribute in Zigbee applications is the provision for over-the-air (OTA) updates. Such updates enable manufacturers to introduce new functionalities, rectify flaws, and roll out security enhancements in response to emerging threats. Yet, if the OTA update protocol lacks sufficient protection, or if the device manufacturer fails to utilize all the built-in safety measures, it could pose a potential security risk.

## 7. ZigBee Link Keys Use-Cases

### 7.1. Preconfigured Link Keys

The policy of how new devices are handled and if they need a preconfigured link key to connect within the network is dictated by the trust center, and in this case if a device does not have a configured link key, it will not join the network.

**Figure 6:**
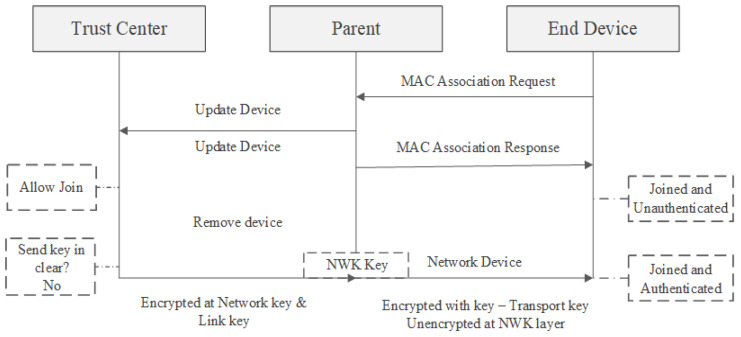
Process of joining a network using a Preconfigure Link Key [23].

The trust center can decide to use either the widely recognized default link key (ZigBeeAlliance09) or a previously shared installation code key. The following diagram depicts the device joining procedure using a preset key. To incorporate a device into the network, the trust center sends the network key encrypted using the device’s preset link key. The process of joining a network with a preconfigured key is presented in Figure 6.

The trust center’s decision to use either a well-known key or an installation code hinges on striking a balance between user convenience and security. Utilizing a well-known key facilitates smoother device integration with minimal user involvement. Nevertheless, using this key to encrypt the network key presents a brief window of vulnerability until the well-known key is substituted with a fresh one. Opting for an installation code ensures more secure initial transmission of the network key to the device, but it demands additional engagement between the user and the trust center [22].

### 7.2. New Link Key Request after Joining

**Figure 7:**
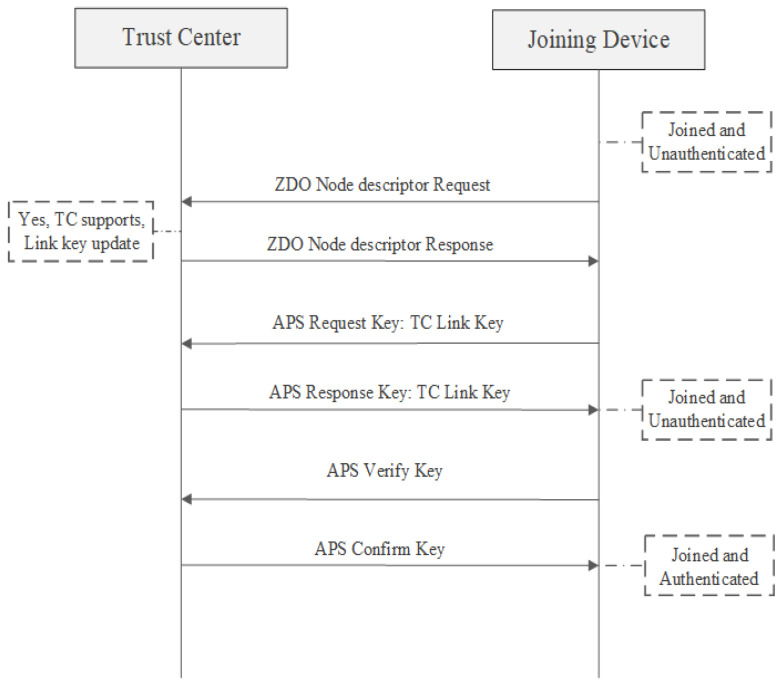
Update the Trust Center Link Key after rejoining [23].

In Zigbee 3.0, devices must ask for an update of the trust center link key once they have successfully joined the network. This new key will supersede their prior preset key and will remain in use as long as the device is connected to that particular network. Regardless of whether the device initially used an installation code key or not, this key will be updated. Trust center link key update process is presented in Figure 7 [22,23].

### 7.3. Secured Rejoining in the Network

**Figure 8:**
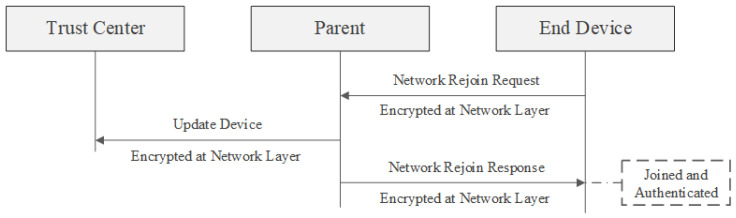
Process of device to securely rejoin the network [23].

A secured rejoin is the primary method a device should use when trying to reconnect to the network. It is straightforward and allows the device to quickly re-establish communication if it has the current network key. This process is especially needed when a mobile or sleepy end device loses its parent connection. As demonstrated in the next figure, the device encrypts and sends its rejoin request using its version of the network key. If a nearby router has the matching network key, it sends an encrypted rejoin response. The device is then authenticated and reconnected. The router that responds informs the trust center of the device’s rejoin, but the trust center does not need to take additional steps. Network secured rejoining is presented in Figure 8.

### 7.4. Secured Trust Center Rejoin

A trust center rejoin becomes essential when nearby devices have transitioned to a new network key and no longer share the same key as the device trying to rejoin. For this rejoin process to work, the device needs to possess a trust center link key. The device forwards an unencrypted rejoin request. A close-by router receives this unencrypted request and sends a response, permitting the device to move to a state where it is connected but not authenticated.

**Figure 9:**
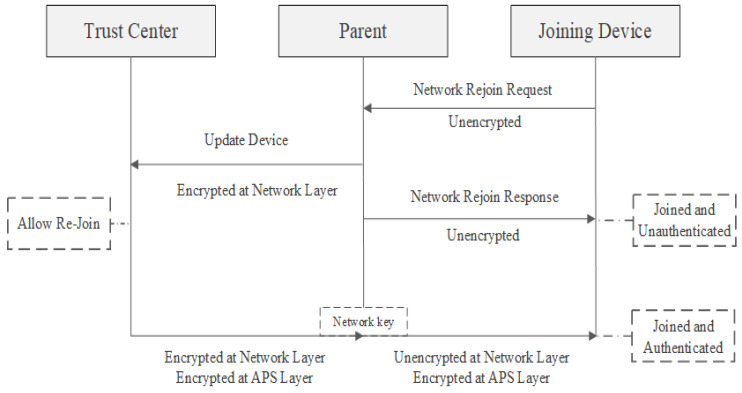
Trust Center secured rejoining process [23].

As depicted in Figure 9, the rejoining device’s parent sends an ‘Update Device’ message to the trust center, alerting it about the unsecured rejoin attempt. The trust center then faces two options: either approve or reject the rejoin. Should it give the green light, an updated network key must be sent to the device. This message, encrypted with the device’s trust center link key, is protected at both the network and APS layers. The parent receives and subsequently passes on this message to the rejoining device, omitting the network encryption. Once equipped with the new network key, the device attains a state of being connected and authenticated, allowing it to re-engage with the network [23].

### 7.5. Trust Center Network Decision Process

**Figure 10:**
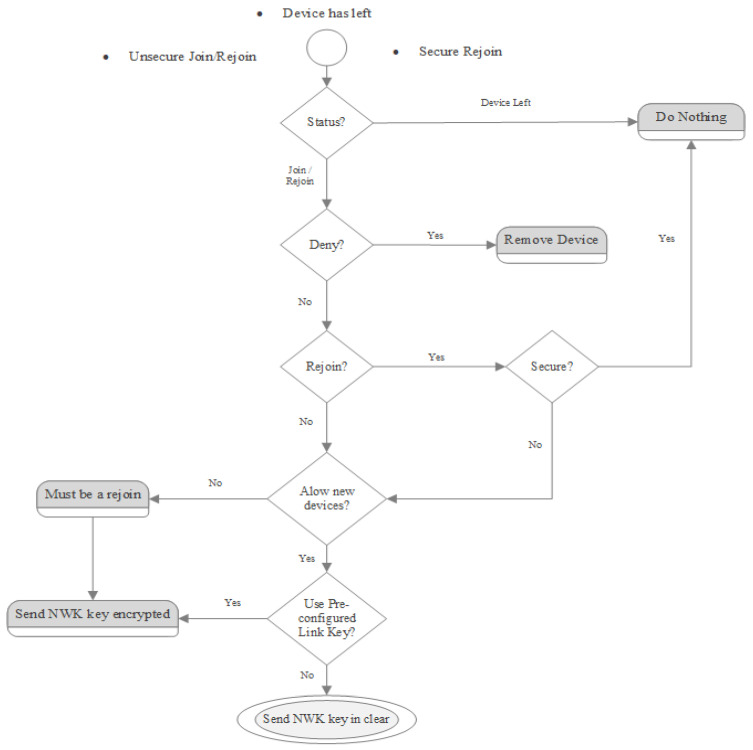
Decision-making process in a Trust Center formed network [23].

The upcoming diagram (Figure 10) showcases the trust center’s decision-making process when a device connects to the network. The parent of a device, whether it is joining anew or rejoining, communicates an ‘Update Device APS’ command to the trust center to signify the occurrence. The trust center application then determines the next course of action based on this information. This representation details the procedure for a Zigbee PRO device integrating with a Zigbee PRO network under standard security protocols.

The trust center holds the authority to determine if devices should be permitted into a Zigbee network and whether to transmit the key transparently. Various factors, such as user actions (such as pressing a button), time-specific conditions, the IEEE address of the device attempting to join, or specific conditions (e.g., the network undergoing commissioning), can influence the trust center’s choice.

When new devices attempt to join, the trust center determines if a preset key should be associated with the device. Joining devices lack the capability to notify the trust center about their possession of a preset key through the Zigbee protocol [23].

## 8. Experimental Results

### 8.1. Network Formation and Experimental Intercepting Set-Up

**Figure 11:**
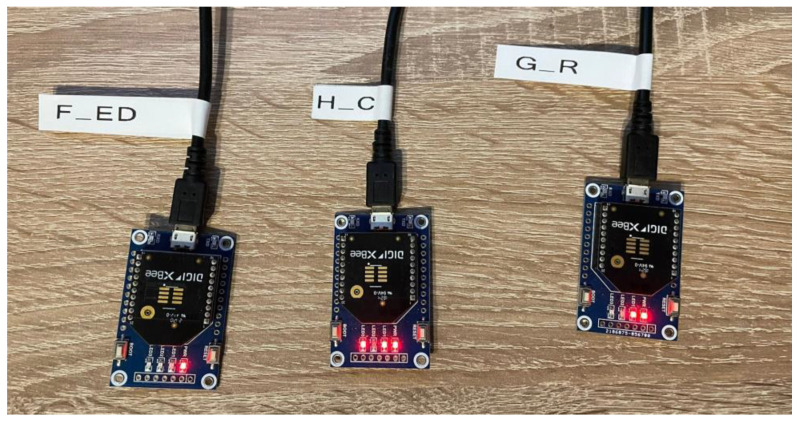
DIGI XBee 3 devices.

In order to send messages between devices, the network has to be formed first. The network is composed of a coordinator, a router, and an end device in order to fulfill needs for both the centralized and distributed security model. Devices used for this experiment are DIGI International Xbee 3 (XB3-24, DIGI International, Ismaning, Germany), based on Zigbee protocol, presented in Figure 11, with the latest firmware of the function set Digi Xbee 3 Zigbee 3.0 TH. Devices configuration for network forming is carried out in XCTU tool, setting the following parameter, ID (extended PAN ID) = 2023, ZS (Zigbee Stack Profile) = 1, which corresponds for Zigbee 2006, DL (Destination Address Low) = FFFF set for broadcast address for the PAN, SC (Scan Channels) = 0x1000 to allow coordinator and devices to connect only using channel 17 and AP (API Enable) = 1 to configure devices in API mode without escapes. This configuration is used for every device in the network together with role configuration.

**Figure 12:**
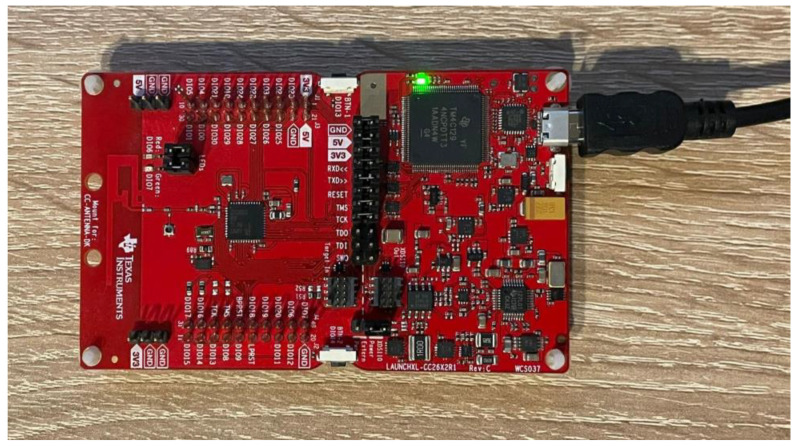
Texas Instruments LaunchXL-CC26X2R1 intercepting kit.

Zigbee is a wireless communication protocol, and, like any wireless protocol, the data packets transmitted over the air can be intercepted or “sniffed” by an unauthorized device within range that is equipped with the appropriate hardware and software. In order to analyze vulnerabilities, a test bench to intercept Zigbee messages was set-up with Texas Instruments LaunchXL-CC26x2R1, presented in Figure 12, Texas Instruments Sniffer Agent and Wireshark application.

In order to begin with intercepting Zigbee traffic, a sniffing board needs to be prepared. In this direction, we flashed the CC2642R wireless MCU with sniffer_fw.hex, a sniffing program available with SmartRF Packet Sniffer 2 application provided by Texas Instrument (Dallas, TX, USA). This hex file was flashed through SmartRF Flash Programmer 2, also provided by TI. After flashing the MCU, in order to collect data from development board, a pipe was configured together with operating channel, 0x17, and radio configuration IEEE 802.15.4 from SmartRF Sniffer Agent application. Because Wireshark application was used to collect data, we imported the pipe previously configured from TI application directly in Wireshark by creating a shortcut of this application and adding in its properties at target field the following command: “C:\Program Files\Wireshark\Wireshark.exe” -i\\.\pipe\tiwspc_data -k. At this point configuration was performed, and intercepting was started by starting SmartRF Sniffer Agent to capture the traffic and opening Wireshark application from the shortcut created to list the packets. An important feature provided by Wireshark is setting-up the key used in Zigbee security, automatically decrypting intercepted packets, which has helped in understanding methods of establishing and transporting security keys.

Using this set-up, we were able to capture all the packets transmitted on this channel with disadvantage of capturing also the packets transmitted by other devices than the ones configured in our network. One of the limitations observed of this set-up is that we were able to intercept packets sent under IEEE 802.15.4 format but without the ability to parse the messages in useful information.

Table 2 shows the security models in which the network was configured, thus establishing the conditions under which the tests were performed. These conditions being based on the security methods defined by the Zigbee Alliance and tested on Digi International Xbee Pro devices to determine and evaluate how the security is achieved.

### 8.2. Isolated Devices Messages

In order to obtain an overview of the messages specific to each type of device in the network, they have been intercepted independently and thus we could observe that the Xbee Pro modules comply with the Zigbee specification when they are not part of any network.

Coordinator messages—In order to understand how Zigbee network works, Zigbee devices were isolated, and the traffic generated by them was analyzed. In this case, the coordinator is sending the following messages: many-to-one Route Request, used to signal a route by the coordinator, to avoid flooding the network with route discovery messages sent by each sensor device that is associated with the private ZigBee network, Link Status, which is an integral part of the network’s routing mechanism and its primary function is to provide devices in the network with up-to-date information about the link quality and the network’s topology. The coordinator also sends a Network Address Request, which is used to retrieve the 16-bit network address of a device on the network, given its 64-bit extended address (or vice versa). This mechanism is essential since while the 64-bit address is globally unique and typically tied to a device’s hardware (similar to a MAC address in Ethernet networks); the 16-bit network address can be assigned dynamically when a device joins a Zigbee network and can change under certain conditions.

Router messages—The coordinator routers send in the network not only Link status messages, with the same purpose as the device mentioned before, but also Beacon requests.

End devices—as routers, end devices are sending in the network Beacon requests, when a device wants to join a Zigbee network, it can scan for beacons from the coordinator or routers; by evaluating the received beacons, devices can choose the best parent device to join based on signal strength or other metrics. When a Beacon message is not sent in the network, devices can send Beacon requests messages in the network.

### 8.3. Message Exchange in a Not Secured Network

**Figure 13:**
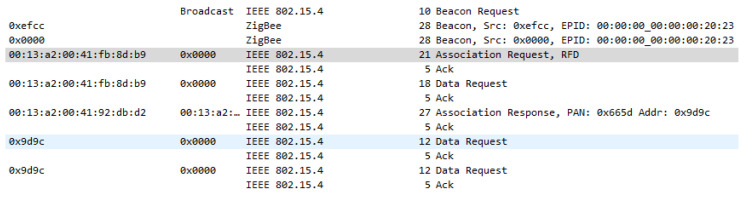
Network formation message exchange—Unsecured Network.

In a network which is not secured neither at the Network layer nor at the Application support layer, the message exchange is performed without security, and the data which are sent through the network are sent unencrypted. When the network is formed, the joining device sends a Beacon request message, while the coordinator responds with a Beacon message followed by an Association request and Data request coming from the joining device. If the joining device has the right parameters, it can join the network and the coordinator sends an association response packet, containing the acceptance status of the new device. Transmitted messages within the network formation sequence are presented in Figure 13.

**Figure 14:**
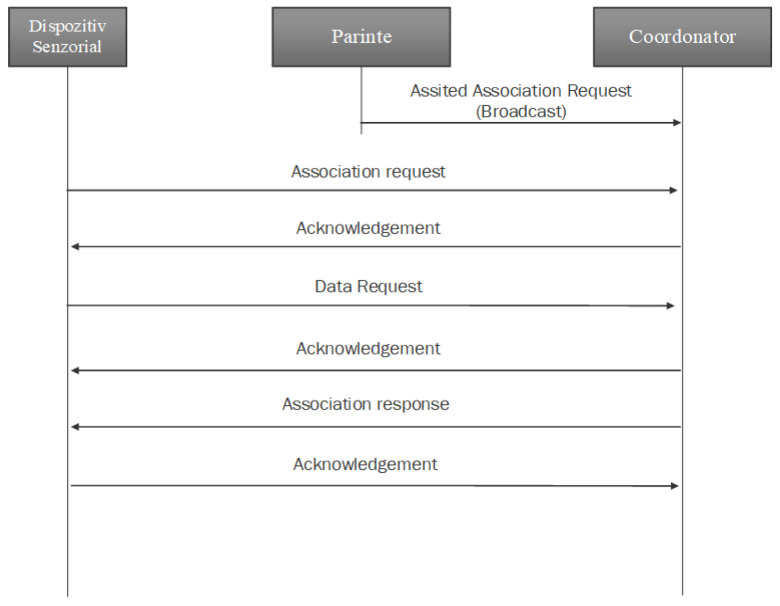
Association message exchange of a new device in the network [23].

The process of association of a new end device/router in the network respects the method presented in Figure 14, which represents the Zigbee specification process, and demonstrated by the intercepted packages from Figure 13.

**Figure 15:**
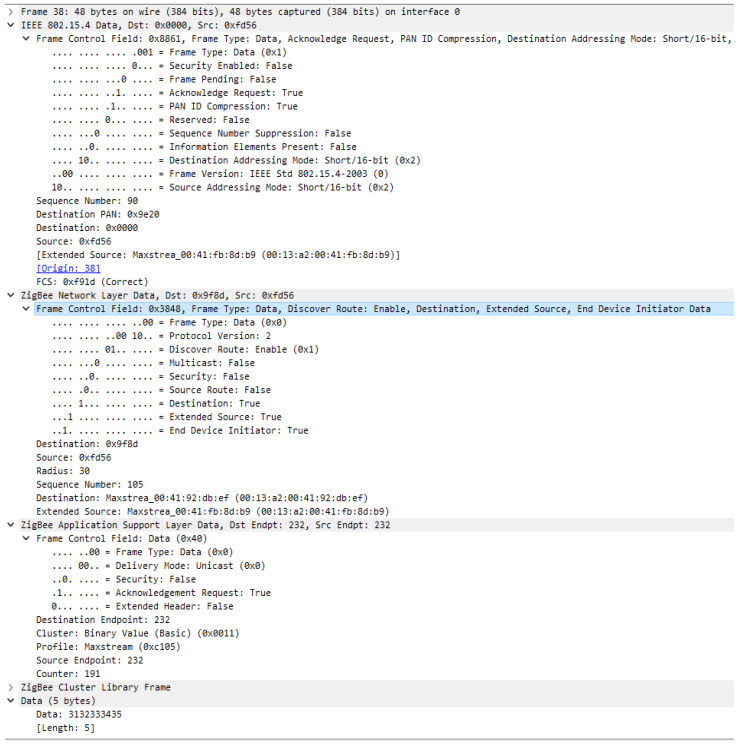
Unsecure message transmission frame format.

In order to understand the security features provided by the Zigbee protocol, an analysis of a not secured message between devices within the same network was performed. Message components are presented in Figure 15, for a frame sent from an end device to a router within the same network formed at Section 7.1.

Starting the analysis from IEEE 802.15.4 standard, we can observe that Security Enable field from the Frame Control component is set to 0 so the security features, such as MIC, encryption, and Frame Counter value at the MAC layer, are not used, which leads to the Auxiliary header not being present. In the MAC header we can only find the following information: configuration of the transmission as frame type, type of addressing for both source and destination, destination PAN, extended source address, etc.

Moving forward to the next layer, this time for a Zigbee stack layer at the Network layer, we observed the same pattern as in MAC layer where the Security field from the Frame Control component is set to 0, leading to the lack of encryption of the message, message integrity code and frame counter value.

At the Zigbee Application Support layer, security is not enabled either, having the same behavior as presented at the previous layers. In order to check all the possibilities of the security, a security transmission was forced but the message was not sent to the destination devices, responding to the request with the following message: Delivery status—2E (Attempted unicast with APS transmission, but EE = 0). The message returned by the devices signals that Security is not activated, the configuration option of the devices being zero, where zero means Network encryption disabled.

### 8.4. Message Exchange in a Distributed Security Model Network

Distributed security model, in Xbee devices, can be achieved in two ways: by enabling device configuration option for using the security only at Network layer with Network key, in this way providing only hop-by-hop encryption and security of the packets transmitted, and by using the Network key together with the global Link key offering possibility of the end-to-end secured transmission of the packets. The distributed security model does not require the presence of a trust center in the network, routers being able to form the network and distribute a copy of the network key to the joining devices.

The configuration for the security model mentioned above was performed by changing devices options to the following configuration: EE (Enable security) = 1, EO (Encryption Option) = 0, which is the default value and corresponds in DIGI Xbee devices to distributed security model, this parameter having the possibility of providing multiple options for security, but these will be discussed in the upcoming sub chapters.

**Figure 16:**
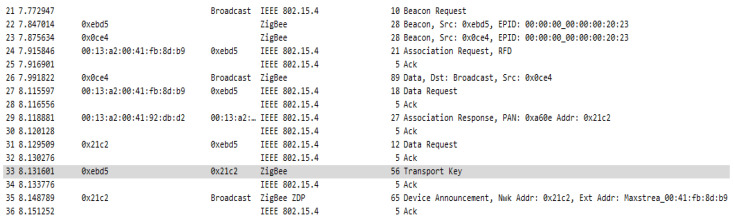
Association sequence in a distributed security model network.

For the first case, where the distributed security model was implemented, additional to the configuration presented before, NK (Trust Center Network Key) = 0000005A69674265654B657932303233 has been updated with the network key value and for KY (Link Key) = 0 which means the link key is not used. In this case at network formation, the same association message exchange is performed as presented in Figure 13 with an additional step, key transport, where routers are sending a copy of the network key, in clear text, to every device that is joining the network. Association sequence is shown in Figure 16.

**Figure 17:**
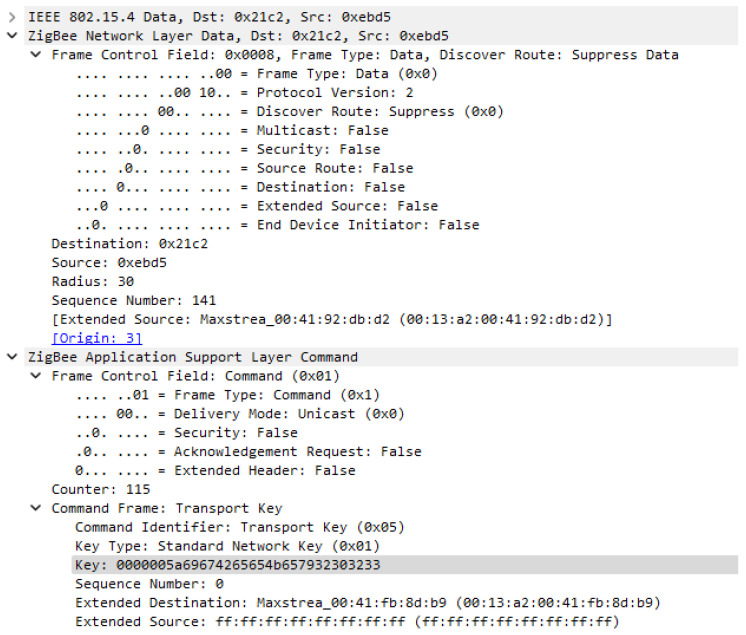
Key transport frame format unencrypted.

The transport key frame has the general frame format, with information about addressing, type of addressing, security that is not enabled, and it is generated at APS layer, as a command frame, in order to send the key to the newly joined device. As we can see in Figure 17, there is no security applied to this frame, neither at network layer nor at application support layer, making this frame to not be protected with any of the security features which Zigbee protocol can provide. Basically, the network key is transmitted unencrypted and if an attacker can force a reset of a router, he can sniff the key during the re-formation of the network and connect to the network and send messages to flood the connections, or send erroneous data to the connected devices, in this case performing a man-in-the-middle attack.

**Figure 18:**
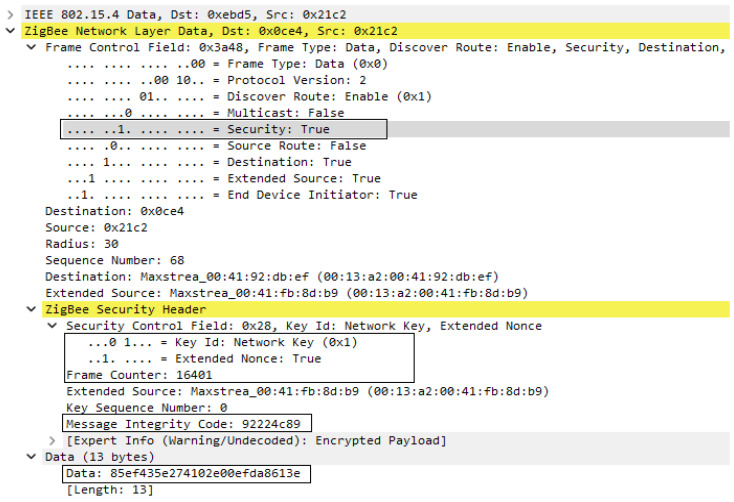
Encrypted Data Frame format.

As regards the data messages transmitted in the network between devices, they were encrypted with the network key, which can also be seen in Figure 18, where we find the Security field active at the level of the Frame Control component for the Network Layer. This time, in addition to unencrypted data frame format, we have the Zigbee Security Header, which contains the Frame Counter and Message Integrity Code fields, the latter being calculated based on the network key. Using the model presented above, the data are secured, being encrypted, authenticated, and protected against replay attacks.

**Figure 19:**
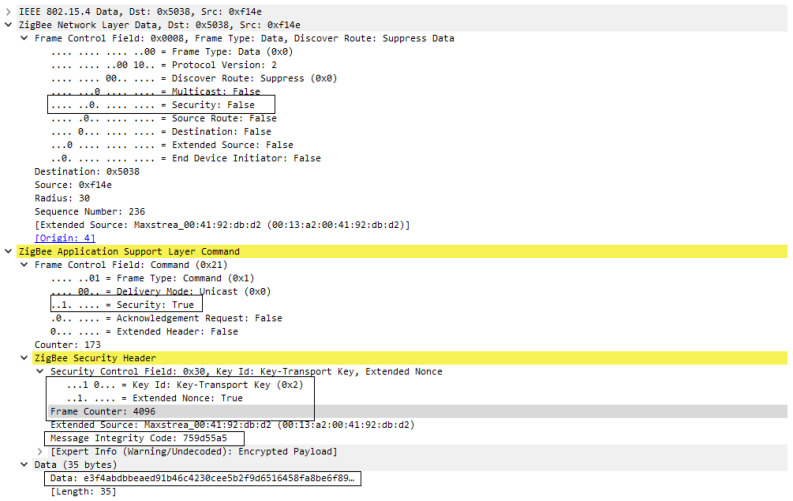
Transport Key frame encrypted with Link Key.

The second solution for the distributed security model involves configuration of a global link key, this being specific for DIGI XBee devices, and can be performed by configurating KY (Link key) = 0000000000000000004C696E6B4B6579 where the value is actually the Link Key. As presented in the first case, the association sequence will be the same as in Figure 16, having the same message exchange, but, in this case, the Transport key frame will be encrypted and authenticated by the link key at the application layer support. So, the Network key is transmitted to the joining device encrypted with the global Link Key. This process is presented in Figure 19.

**Figure 20:**
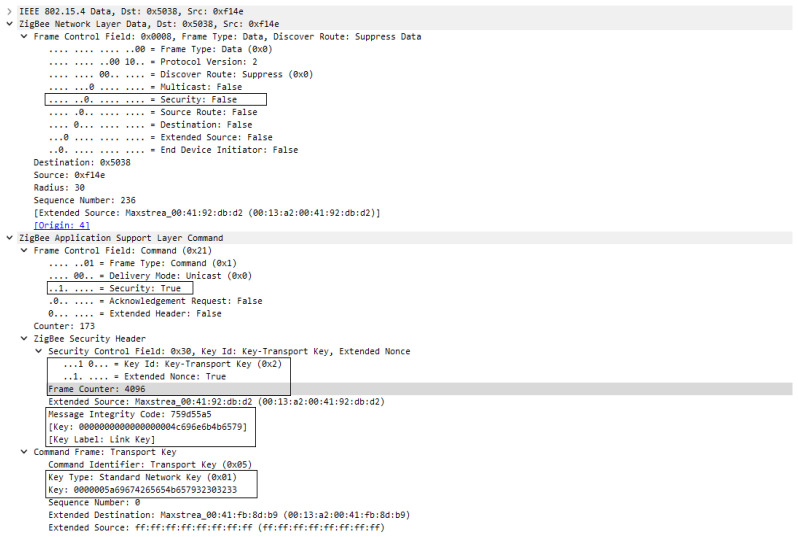
Transport Key frame encrypted with Link Key—decrypted at destination.

In both Figure 19 and Figure 20 we can see that security is active only at the Application support layer; this is happening because the network key is not yet set for the new devices and the Transport key frame is encrypted by the Link key which is used only at APS layer. In Figure 19, where the frame was decrypted, we can see the transported key is Standard Network Key and its value is 0000005A69674265654B657932303233.

**Figure 21:**
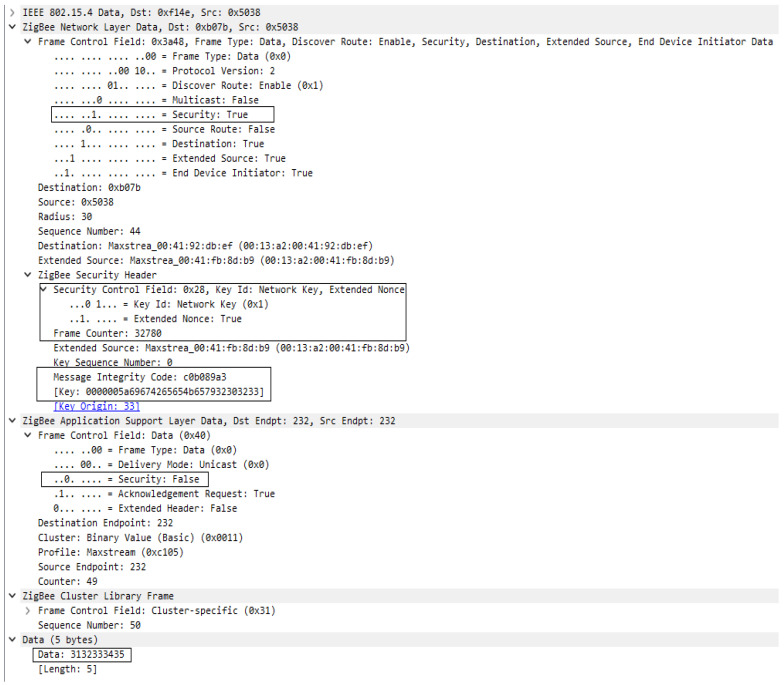
Data packet encrypted only with network key.

**Figure 22:**
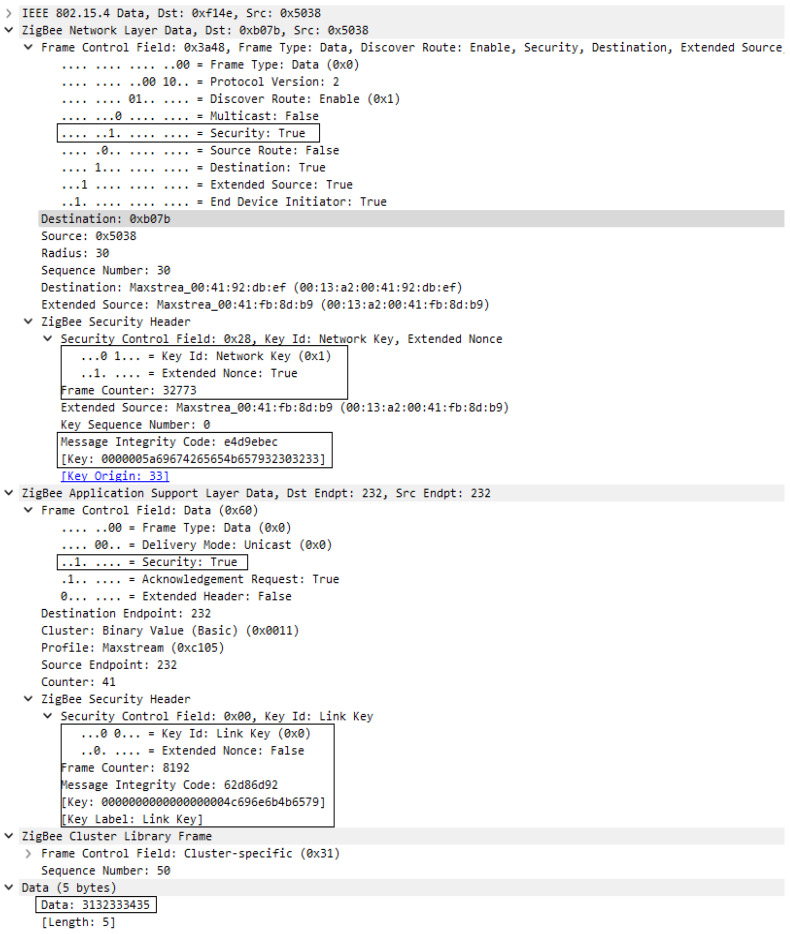
End-to-end secured data frame format.

In this security model there are two options for sending messages between devices: encrypted by Network key only or encrypted end-to-end, where data are encrypted first with the Link Key and after with the network key. In Figure 21, hop-by-hop security has been applied and in Figure 22 end-to-end security. Wireshark offers the possibility of registering keys that are used within the network and automatically decrypts the messages with the introduced key, those being signaled inside the security header of each layer frame.

In Figure 22 we can see full security features on a transmitted data frame, having encryption, authentication, and protection against replay attacks at both Zigbee stack layers, Network layer, and Application support layer. The first level of security is applied at the Application support layer, which is the highest layer in the stack, and, based on the Link key, payload is encrypted, frame counter is applied to frame, and all this information is authenticated using the same Link key resulting in a 4-byte message integrity code. All this information grouped in one string of bytes represents the APS frame which goes to the next layer of the Zigbee stack, the Network layer, where, using the same algorithm, AES-128, APS frame is considered to be Payload, and it is encrypted. After the encryption frame counter is added, together with the header, security header, and payload, it forms the Network layer frame. The network frame is authenticated using the network key this time, and the same algorithm, AES-128. The security of the two layers can work independently of each other.

### 8.5. Message Exchange in a Centralized Security Model Network

A network secured by centralized security mode is based on a trusted center that is part of the network and around which this security is built. Even if the security method is centralized, all network topologies can be used with it. To be able to operate in this security model the devices must be configured accordingly as follows: In addition to the previous model the EO parameter is configured with the value 2, this value corresponds to the centralized trust center usage model.

**Figure 23:**
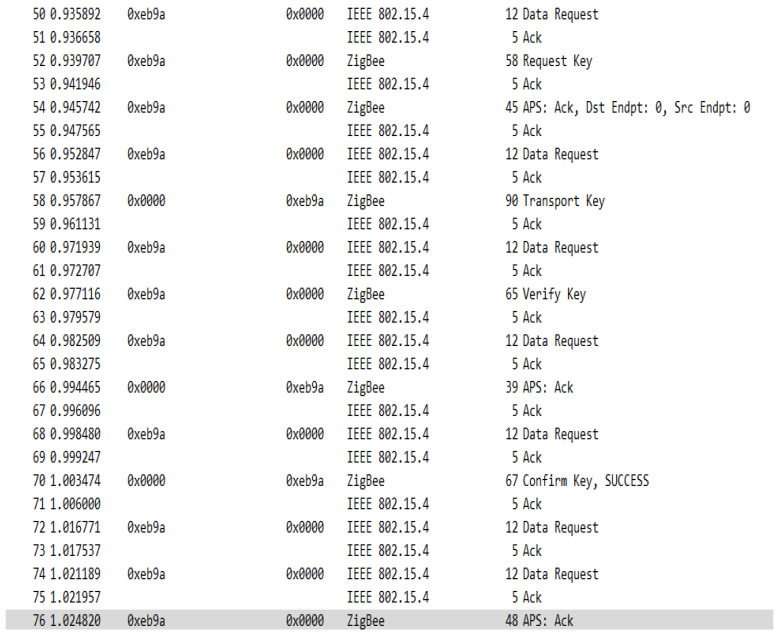
Centralized network device association message sequence.

In the case of this configuration when forming the network, the association of devices is carried out based on the preconfigured global link key, the network key is transmitted via the transport frame to the device requesting the association, but this time, this transport is encrypted with the preconfigured global link key. In addition to the classical key transport messages, corresponding to the previous model, in the case of the centralized model when associating a new device, several key transport messages are observed containing a device-trust center link key which is established by the trust center and is also transmitted to the associating device. In addition to these messages, messages verifying this key can also be observed. The sequence of messages when associating a device to the network is shown in Figure 23.

**Figure 24:**
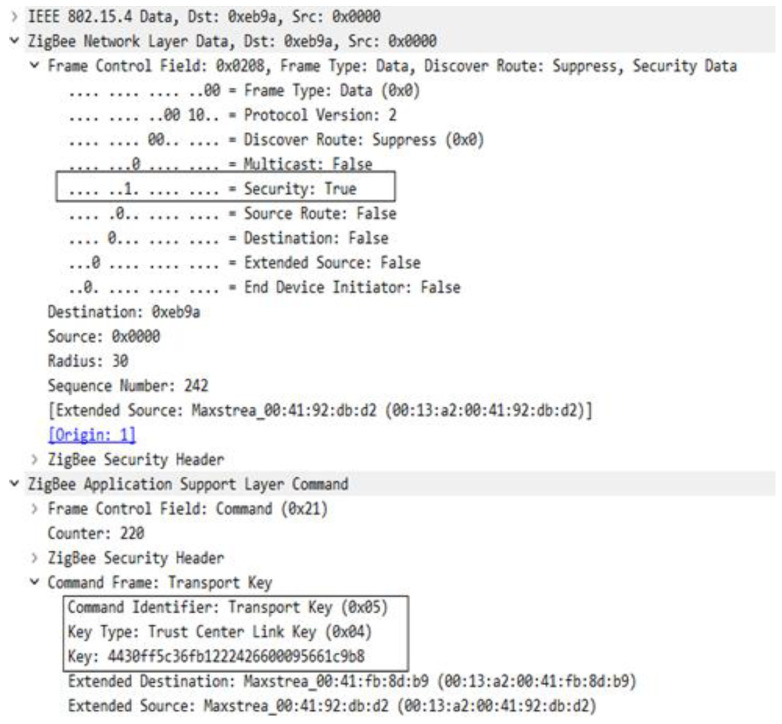
Key transport frame format of link key with trust center.

In the sequence in Figure 23, you can see the messages used to transport the keys, but also the messages sent with the intention of verifying the link key with the trust center. After the key is transmitted a message—Verify Key is sent to the device, the device then transmits a packet of data encrypted by this key, which is then verified by the coordinator, and if everything is OK the Confirm Key frame is transmitted and thus the device is officially joined to the network. Figure 24 shows the structure of the Transport Key frame for the Link Key with the Trust Centre.

**Figure 25:**
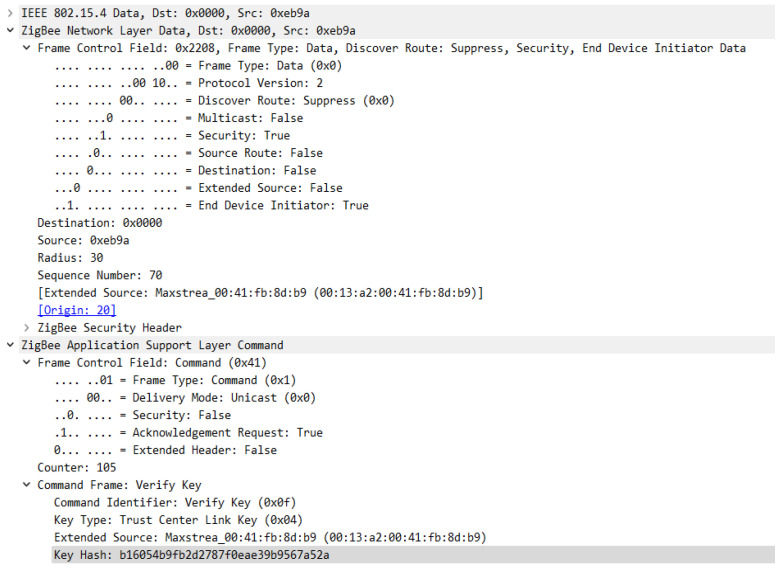
Verify Key Frame Format.

**Figure 26:**
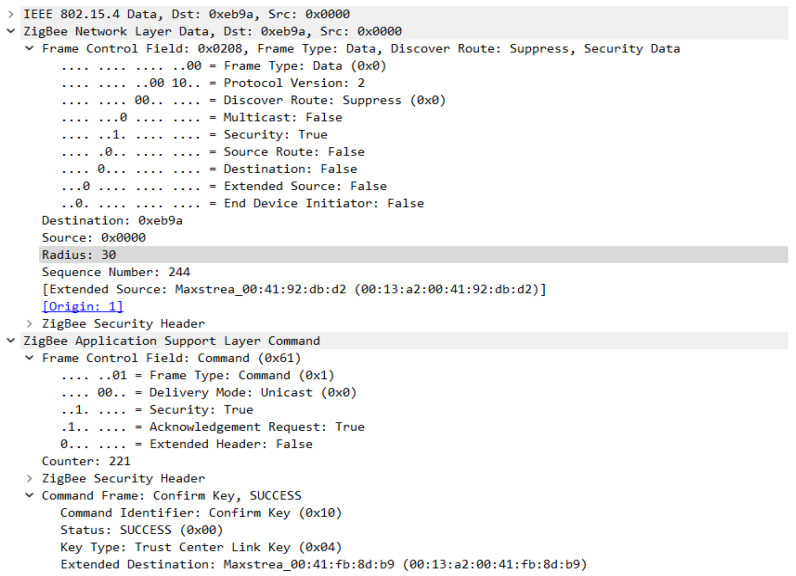
Confirm Key Frame Format.

Figure 25 and Figure 26 show the format of the Verify Key and Confirm Key frames, these messages are used to confirm the transport of the trust center link key to the device attempting to join the Zigbee wireless communication protocol-based sensor network.

The objective of this study is to evaluate the security models of Xbee devices for network formation and network messaging in comparison to the models defined by the Zigbee Alliance. In our experiments, we were able to observe the security methods used by these devices, in accordance with the Zigbee specification methods. The results obtained are shown in Table 3.

Based on the experimental results obtained and the processes observed during the experiments, I can evaluate the security of Xbee Pro 3 devices. Given that during the formation of the network, the network key is transmitted either in plain text or encrypted using the link key through Xbee devices, and considering that the link key is general and preset in all these devices, I concluded that the security measures in Xbee primarily revolve around the knowledge of the link key. Possessing knowledge of the link key and being a part of the network formation process allows for the detection of the TC link keys and network key, thereby creating a vulnerability in these devices for attacks as man-in-the-middle or reply. These attacks target unauthorized network access and data integrity by altering data as they are routed, and by replaying messages previously transmitted by authentic devices.

One of the limitations of this study is that the devices were only configured from the XCTU application, but they can also be configured via radio commands. By investigating configuration via radio commands, it can be studied whether security can be used to the full potential offered by the Zigbee specification. Another limitation is the software loaded on the development board developed by TI. As it is not open-source, it cannot be evaluated, whether it performs well or not, as it has no reviews.

Note that the devices used to form the network were Xbee 3 (Xb3-24), no other Zigbee devices were part of the network. From the theoretical analysis performed on Xbee modules in general we could observe that they can use the latest firmware versions available, but this is not enough to be able to generalize this study to all Xbee modules.

As technology advances, the security of Xbee 3 devices continues to be a priority. Future research could explore in depth the application of digital signatures, using algorithms such as RSA or ECDSA, to improve the security of these devices. Such approaches could provide a higher level of authentication and data integrity, protecting devices against attacks and ensuring the safety of the information transmitted. Digital signatures ensure that the message or data really comes from the declared source, which helps prevent man-in-the-middle attacks and other types of fraud. Moreover, any changes to the data after they have been digitally signed will result in an unsuccessful signature verification, thus guaranteeing the integrity of the data transmitted over the network together with authentication mentioned before. The digital signature system enables centralized key management and scalability, making it easy to add new devices to the network without compromising security. Adopting and optimizing these encryption technologies could also facilitate the deployment of scalable and adaptable security solutions that meet the evolving needs of Xbee Pro-based networks.

## 9. Conclusions

This study examines the Zigbee protocol along with its security features provided at the architecture layers. The study presents the configuration of the network, the setup of the test bench, the definition of the test conditions, the test execution, and the analysis of the experimental results. All these elements being implemented to evaluate the security methods encountered in Digi International’s Xbee 3 devices. The study also includes the limitations identified in the workbench and test methodology, taking into account the characteristics of the Zigbee specification.

From the results we obtained, we were able to validate the basic principles and methods by which Xbee modules operate in the network in specific situations such as:

- Network formation;
- Connecting a device to the network;
- Key exchange between devices (network key and link key);
- Data encryption hop-by-hop or even end-to-end.

Moreover, we invalidated the theory in which the TC establishes link keys between two devices (other than TC) and the mechanism by which the TC establishes and distributes these keys, which is in contrast with Zigbee specification. The link key between devices is general and preconfigured on devices before network formation during configuration of the devices by weakening device security. The link key between TC and devices is established upon association; it cannot be preconfigured, but only generated based on certain parameters, such as address or install codes. Based on the fact that during network formation, through Xbee devices, the network key is transmitted in plain text or encrypted with the link key, and that the link key is general and preconfigured in all these devices, I came to the conclusion that security features in Xbee come down to knowing the link key. By knowing the link key and participating in the network formation process, the TC link keys can be detected, and security is no longer available in the network.

To avoid security issues, before transmitting sensitive data, device authentication could be performed through asymmetric encryption algorithms, specifically using digital signature algorithms. On the other hand, if the link key is kept secret, the security mechanisms of Xbee devices can be very efficient.

## Figures and Tables

**Table 1:** IEEE 802.15.4 protocol security options [16].

Security Level	Security Attributes	Data Confidentiality	Data Authenticity	MIC Length	Replay Protection
000	Null	OFF	NO	0	YES
001	MIC-32	OFF	YES	4	YES
010	MIC-64	OFF	YES	8	YES
011	MIC-128	OFF	YES	16	YES
100	AES-CTR	ON	NO	0	YES
101	ENC-MIC-32	ON	YES	4	YES
110	ENC-MIC-64	ON	YES	8	YES
111	ENC-MIC-128	ON	YES	16	YES

**Table 2:** An overview of network security model configurations used for experiments.

ID	Experimental Conditions
1	Unsecured Zigbee mesh network
2	Zigbee mesh network configured based on distributed security model with network key
3	Zigbee mesh network configured based on distributed security model with network key and link key
4	Zigbee mesh network configured based on centralized security model with network key and link key

**Table 3:** Experimental results comparing Xbee and Zigbee Alliance devices.

Xbee Pro 3	Zigbee Alliance
The network key is shared to the Xbee devices in clear text, if the link key is not configured, in the joining process of a new node.	The network key is shared to the Xbee devices in clear text, if the link key is not configured, in the joining process of a new node.
The network key is shared with the device that joins the network encrypted with the application link key.	The network key is shared with the device that joins the network encrypted with the application link key.
The application link key is generally configured manually for all devices and is not configured between pairs of devices as in the Zigbee specification.	Application link keys can be configured manually or can be set by the trust center between all pairs of devices communicating with each other. If a device on the network wants to communicate securely with another device on the network, it makes a request for the binding key to the trust center, and the trust center sends the devices the binding key encrypted with the trust center binding key, a process not available in Xbee devices.
The TC link key is established between devices when a device joins the network. The key is transmitted encrypted with the configured global application link key.	The Trust Center holds the capability to determine the management of its TC link keys. It has the discretion to opt for distinct keys for every device in the network, keys that are derived from a shared data point, or a universal key applicable to all devices in the network. Additionally, the negotiation of Trust Center link keys can occur at the application layer through the utilization of a key establishment protocol, such as Certificate-Based Key Establishment (CBKE).

## Data Availability

Not applicable.
